# Individual and combined anti-trypanosomal effects of arteether and diminazene aceturate in the treatment of experimental *Trypanosoma brucei brucei* infection in rats

**DOI:** 10.14202/vetworld.2020.1858-1862

**Published:** 2020-09-11

**Authors:** Tobias Nnia Egbe-Nwiyi, Samson Eneojo Abalaka, Nuhu Abdulazeez Sani, Oremeyi Zainab Tenuche, Idoko Sunday Idoko

**Affiliations:** Department of Veterinary Pathology, Faculty of Veterinary Medicine, University of Abuja, Abuja, Nigeria

**Keywords:** albino rats, arteether, clinicopathology, diminazene aceturate, *Trypanosoma brucei brucei*

## Abstract

**Aim:**

Trypanosomosis is a vital protozoan disease of man and animals with devastating consequences in the tropical parts of the world, necessitating the investigation of the effects of diminazene aceturate (DA) and arteether (AR) on *Trypanosoma brucei brucei* experimental infection in rats.

**Materials and Methods:**

We used a total of 98 rats, which were divided into 14 groups (A-N) of seven rats each over 36 days after acclimatizing them. We administered 1×10^6^ trypanosomes to the infected groups (B-N) with Group A as the unexposed control rats. Groups C-F became the infected and treated rats with 3.5 mg/kg, 7.0 mg/kg, 10.5 mg/kg, and 14.0 mg/kg of DA while Groups G-J became the infected and treated rats with 0.01 ml/kg, 0.02 ml/kg, 0.03 ml/kg, and 0.04 ml/kg of AR. Groups K-N became infected and treated rats with DA and AR combinations at similar doses.

**Results:**

Parasitemia suppression occurred in Groups G-J only but became cleared in Groups C-F and K-N. Survival time varied significantly (p<0.05) between Group B and the other infected groups. We recorded anemia in all the infected rats while significant (p<0.05) splenomegaly and hepatomegaly occurred in Groups G-J only compared to the other groups.

**Conclusion:**

AR did not inhibit or potentiate the anti-trypanosomal efficacy of DA, and therefore, it is comparatively less effective in combating *T. brucei* infection at the present doses and treatment regimen.

## Introduction

Trypanosomosis is a serious tsetse fly-borne protozoan parasite disease that affects humans and animals [1]. African animal trypanosomosis reportedly causes poor milk yield, poor hair coat, emaciation, anemia, anorexia, intermittent fever, lethargy, infertility, ascites, abortion, and death of the affected animal if it is not correctly treated [2]. There are many species of trypanosomes, but the dominant pathogenic species affecting animals are *Trypanosoma vivax*, *Trypanosoma* *congolense*, *Trypanosoma simiae*, *Trypanosoma godfreyi*, and *Trypanosoma brucei brucei* [3]. *Trypanosoma* brucei *rhodesiense*, and *Trypanosoma* brucei *gambiense* cause human African trypanosomosis or sleeping sickness [4]. However, *T. brucei* affects tissues mainly. The control of animal trypanosomosis has become a major problem as the parasites exhibit antigenic variation, thereby posing a significant threat to livestock vaccination [5]. There is also drug resistance in some cases of animal trypanosomosis [6]. The use of trypanotolerant animals, chemotherapy, and tsetse control is some of the ways advocated for the elimination of animal trypanosomosis [7-9].

Animal trypanosomosis responds to many treatment drugs, among which is diminazene aceturate or DA (Berenil^®^, Hoechst, Germany) as a conventional trypanocide so far although relapses have been reported [6,10]. *Plasmodium falciparum*, including *Plasmodium vivax*, *Plasmodium knowlesi*, *Plasmodium ovale*, and *Plasmodium malariae*, is a protozoan parasite that causes malaria in humans [11]. Therefore, protozoan parasites cause both trypanosomosis and plasmodosis of animals and human beings [12], among others. Drugs such as amodiaquine hydrochloride (Camoquine^®^, Pfizer, Germany), pyrimethamine and sulfamethoxy pyrazine (Metakelfin^®^, Pfizer, Germany), chloroquine phosphate, sulfadoxine and pyrimethamine (Fansidar^®^, Swipha, Nigeria), and artesunate (Rekmal^®^, Lincoln Pharmaceuticals, Ltd., India) have shown effectiveness in the treatment of plasmodosis. However, they are weak in the treatment of trypanosomosis [2,13]. Arteether or AR (Artebeta^®^, Swiss Parenterals, Gujarat, India) is the ethyl ether derivative of dihydroartemisinin [14] as well as a semi-synthetic derivative of artemisinin, which is a natural plant product of the Chinese plant *Artemisia annua* [15]. Artemisinins reportedly inhibit the *in vitro* growth of *Trypanosoma cruzi* and *T. brucei rhodesiense* [16] even as chloroquine-resistant human plasmodosis responds well to it [17].

Despite the reported effects of artemisinins in human trypanosomosis and plasmodosis, little is known about its effects in animal trypanosomosis hence the present study. Besides, the presence of any comparative trypanocidal effects in animals over the conventional trypanocidal DA can easily be exploited for meaningful animal production. Therefore, the essence of this study was to ascertain the anti-trypanosomal effects of AR and DA each and together in the treatment of experimental *T. brucei brucei* infection in rats.

## Materials and Methods

### Ethical approval

We carried out the work as approved by the University of Abuja Ethics Committee on Animal Use with Reference No.: UAECAU/2020/003.

### Study period and location

The study was conducted from January to March 2020 at the University of Abuja. Abuja, Nigeria.

### Experimental animals

We used 98 healthy adult albino rats of both sexes weighing between 180 and 200 g obtained from the Nigerian Institute for Trypanosomosis Research (NITR), Vom, Nigeria, for the experiment. The 2 weeks laboratory-conditioned rats were randomly divided into 14 groups of seven rats, fed with commercial growers mash (ECWA Feeds Ltd., Jos, Nigeria), and housed in clean cages at ambient temperature (30-35°C) and water were provided *ad libitum* throughout the study. The animals were screened for the presence of hemoparasites [18] before the experiment started.

### Trypanosome stock and inoculation

The obtained *T. brucei brucei* (Federer strains) from NITR, Vom, Nigeria, were serial passages in rats to maintain the parasites. We injected 1 ml of phosphate-buffered saline solution (pH 7.4) containing an estimated 1×10^6^ trypanosomes intraperitoneally into each rat in the infected groups (B-N). The rats were examined daily for the presence or appearance of the parasites.

### Test drugs

We prepared 75 mg of AR (Artebeta^®^, Swiss Parenterals, Gujarat, India) and 7% DA (Berenil^®^, Hoechst, Germany) following the manufacturer’s specifications.

### Experimental design

The experiment involved a total of 98 albino rats that we divided into 13 groups (A-N) of seven rats each. We experimentally infected rats in Groups B-N (91 albino rats) with *T. brucei brucei* parasites (1×10^6^ trypanosomes) with Group A, containing the uninfected and untreated control albino rats. We administered both drugs separately, intramuscularly. This process involved the administration of DA once at dose rates of 3.5 mg/kg, 7.0 mg/kg, 10.5 mg/kg, and 14.0 mg/kg to each of the albino rat in Groups C-F and AR, at 0.01 mg/kg, 0.02 mg/kg, 0.03 mg/kg, and 0.04 mg/kg to albino rats in Groups G-J, respectively. Groups K-N were infected and treated with DA and AR separately at similar doses. We treated the infected rats on day 12 post-infection (Pi) while AR treatment in Groups G-N continued on days 13-14 Pi.

### Parasitemia and packed cell volume (PCV) determination

We collected blood samples from the experimental pre-trypanosome and post-trypanosome inoculated rats to determine the level of parasitemia every 2 days by the hematocytometer method. Similarly, we determine the PCV level every 4 days through the microhematocrit method [18].

### Determination of survival time (ST)

We monitored the experimental rat’s daily Pi until the termination of the work (A-N) or until the day of death (B-N, where applicable) to determine the Pi ST for each rat.

### Organosomatic indices determination

We sacrificed the rats at the end of the experimental period (for those alive only). Likewise, we immediately conducted a postmortem examination on each of the carcasses (the sacrificed or dead rats), according to standard procedures [19]. We promptly weighed the harvested liver and spleen, and used them to determine the hepatosomatic and splenosomatic index of the experimental rats, as described by Vani and Reddy [20].

### Statistical analysis

We expressed the data as means ± standard deviations and subjected them to a one-way ANOVA at p<0.05 statistical significance, according to Chatfield [21].

## Results

**Figure-1:**
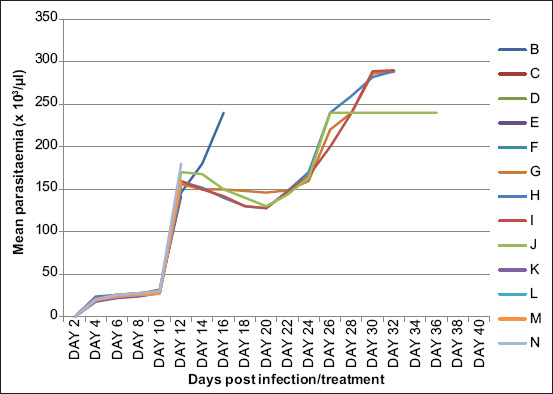
Mean parasitemia of *Trypanosoma brucei brucei* infected untreated rats (Group B), infected and treated with 3.5 mg/kg of diminazene aceturate (Group C), 7.0 mg/kg of diminazene aceturate (Group D), 10.5 mg/kg of diminazene aceturate (Group E), 14.0 mg/kg of diminazene aceturate (Group F), 0.01 ml/kg of arteether (Group G), 0.02 ml/kg of arteether (Group H), 0.03 ml/kg of arteether (Group I), 0.04 ml/kg of arteether (Group J), 3.5 mg/kg of diminazene aceturate, and 0.01 ml/kg of arteether (Group K), 7.0 mg/kg of diminazene aceturate and 0.02 ml/kg of arteether (Group L), 10.5 mg/kg of diminazene aceturate, and 0.03 ml/kg of arteether (Group M) and 14.0 mg/kg of diminazene aceturate and 0.04 ml/kg of arteether (Group N).

We recorded parasitemia in all the infected rats 4 and 6 days Pi (Figure-1) with a mean pre-patent period range of between 4.3±1.0 and 4.6±1.0 days that did not differ significantly (p>0.05) among the infected groups (B-N). However, we observed a gradual rise in parasitemia in the infected rats (B-N) from day 6 Pi until it peaked on day 12 Pi before the treatment commenced.

Groups C-F and K-N became aparasitemic as from day 14 Pi (day 2 post-treatment) until day 36 Pi (day 24 post-treatment) when we terminated the experiment (Figure-1). We recorded a decreased level of parasitemia in Groups G-J from days 14 to 22 Pi (day 9 post-treatment) before rising again from day 24 Pi (day 12 post-treatment). The level of parasitemia increased gradually in Groups G, H, I, and J until day 36 Pi (day 20 post-treatment) when all the rats died. However, we observed suppressed parasitemia in Group J (treated with 0.04 mg/kg Artebeta^®^) from day 28 Pi (day 16 post-treatment) till day 36 Pi (day 24 post-treatment).

**Figure-2:**
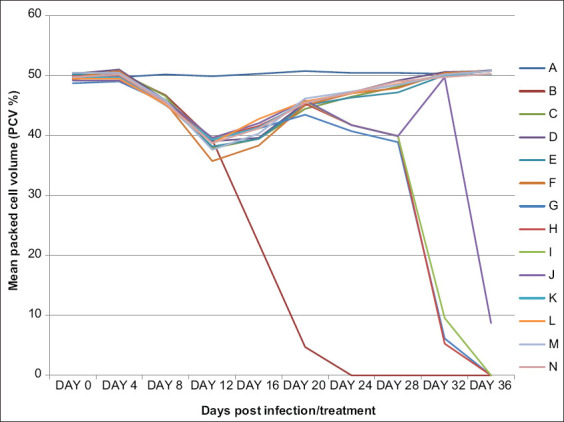
Mean packed cell volume of uninfected untreated rats (Group A), *Trypanosoma brucei brucei* infected untreated (Group B), infected and treated with 3.5 mg/kg of diminazene aceturate (Group C), 7.0 mg/kg of diminazene aceturate (Group D), 10.5 mg/kg of diminazene aceturate (Group E), 14.0 mg/kg of diminazene aceturate (Group F), 0.01 ml/kg of arteether (Group G), 0.02 ml/kg of arteether (Group H), 0.03 ml/kg of arteether (Group I), 0.04 ml/kg of arteether (Group J), 3.5 mg/kg of diminazene aceturate and 0.01 ml/kg of arteether (Group K), 7.0 mg/kg of diminazene aceturate and 0.02 ml/kg of arteether (Group L), 10.5 mg/kg of diminazene aceturate and 0.03 ml/kg of arteether (Group M) and 14.0 mg/kg of diminazene aceturate and 0.04 ml/kg of arteether (Group N).

We recorded a progressive significantly (p<0.05) different mean ST of 18.0±0.0, 27.1±0.2, 30.7±0.9, and 34.4±0.9 in Groups B, G, H, I, and J, respectively. The ST was significantly (p<0.05) shortest in Group B and longest in Group J, comparable to Groups G and H. The PCV decreased significantly (p<0.05) from day 8 to 14 Pi in all the infected Groups B-N compared the control (Figure-2). However, we recorded increasing PCV after that from day 16 Pi (day 4 post-treatment) before returning to their corresponding pre-infection values by day 36 Pi (day 24 post-treatment). On the other hand, the groups treated with different doses (0.01 mg/kg, 0.02 mg/kg, 0.03 mg/kg, and 0.04 mg/kg) of AR showed temporary PCV recovery between days 16 and 20 Pi (days 4 and 8 post-treatment) and started decreasing again gradually. All the infected groups (B-N) showed a comparable level of anemia before and after treatment. However, the anemia became more significantly (p<0.05) severe in Group B (infected untreated) compared to those recorded in the infected and AR-treated rats (Groups G, H, I, and J). We observed significant (p<0.05) enlargement of the liver and spleen of Group B rats compared to the infected and AR-treated Groups G-J (Table-1).

**Table-1:** Hepatosomatic and splenosomatic index of *Trypanosoma brucei brucei* infected and Arteether treated albino rats (n=7).

Parameters	Uninfected untreated control	Infected untreated control	Arteether treatment			

			Infected+ Treatment (0.01 mg/kg)	Infected+ Treatment (0.02mg/kg)	Infected+ Treatment (0.03 mg/kg)	Infected+ Treatment (0.04 mg/kg)
HSI	3.0±0.4^a^	9.6±0.4^c^	5.0±0.8^c^	5.6±0.8^c^	5.5±0.8^c^	4.8±0.9^c^
SSI	1.0±0.4^a^	5.9±0.6^b^	2.9±0.4^c^	2.7±0.7^c^	2.8±0.4^c^	2.9±0.8^c^

Values in rows with different superscripts differ significantly (P<0.05) compared to the uninfected untreated control n=Number of rats, HSI = Hepatosomatic index; SSI = Splenosomatic index

## Discussion

The present study has demonstrated that AR treatment was less effective in the treatment of experimental *T. brucei brucei*-induced trypanosomosis compared to the DA treatment. AR suppressed the level of parasitemia temporarily and prolonged the ST of the infected rats compared to the level of the parasitemia and ST in the infected untreated rats (Group B). The AR suppressed parasitemia observed in this study did not differ from the findings of earlier workers in rats infected and treated with amodiaquine hydrochloride, pyrimethamine, and sulfamethoxy pyrazine, chloroquine phosphate, sulfadoxine and pyrimethamine, and artesunate [13]. The non-clearance of trypanosomes from the peripheral blood of rats infected with trypanosomes and treated with AR could be due to insufficient dose administration because artemisinins have reportedly inhibited *in vitro* growth of *T. cruzi* and *T. brucei* rhodesiense [16].

Diminazene aceturate has shown that it is a reliable trypanoside as it cleared the parasites from the peripheral blood of the treated infected rats (Groups C-F). However, there are reports of observable relapses following infection with different strains of trypanosomes that is treated with DA [6,22]. Similarly, the combined AR and DA administration also cleared the trypanosomes from the peripheral blood of the infected and treated rats with both substances. However, the effectiveness of the combination therapy might be attributable to its DA components rather than to its AR component. Such efficacy might be due to either inhibiting or potentiating the actions of DA at the different doses by AR. The lack of synergism between AR and DA in the present study did not agree with the observations of Egbe-Nwiyi *et al*. [13] in rats infected with *T. brucei brucei* and treated with 2.4 mg/kg of artesunate and 3.5 mg/kg of DA. Nevertheless, AR’s inability to clear the parasite from the peripheral blood agreed with the findings of previous workers who used anti-malarial drugs to treat animal trypanosomosis [2,13].

The observed anemia could have arisen from either host or parasite-derived mechanisms [23]. The host-derived mechanism involves massive host cellular response [23], while the parasite-derived factors involve the release of substances that modify red blood cell (RBC) to enhance their elimination by the host [24]. The reduced PCV is usually associated with the hemolysis-induced acute phase of the anemia [25] due to the host immune response [26]. However, the chronic phase of the anemia is usually associated with hemodilution arising from increased total plasma volume and the low but physiologically relevant immune system-induced RBCs sensitization by the host IgM [26]. Earlier workers have reported similar anemia in animal trypanosomiasis [27]. The gradual recovery of the PCV of the combined AR- and DA-treated rats showed the efficacy of the drugs in clearing trypanosomes from the peripheral blood as earlier reported [6].

Both the acute and chronic phases of trypanosome-induced anemia are known to cause the observed hepatomegaly and splenomegaly [26]. The splenic enlargement might be due to RBCs sequestration [28]. Igbokwe and Nwosu [27] reported similar hepatomegaly and splenomegaly in *T. brucei*- and *Trypanosoma concolense-*infected rats. However, they opined that the liver and the spleen’s mere enlargement did not significantly (p>0.05) contribute to the development of the observed anemia. Besides, the increased hepatosomatic and splenosomatic indices of the infected untreated rats (Group B) indicated the adverse effects of *T. brucei* infection in the exposed rats, especially as the phagocytic activities of these organs are increased [29]. Nevertheless, the observed significant (p<0.05) reductions in the AR- and DA-treated groups compared to the infected untreated control group showed the ameliorative effects of both therapeutic compounds in affected rats.

## Conclusion

AR alone suppressed the level of parasitemia temporarily and prolonged the ST compared to the DA and AR/DA exposed groups, respectively. AR also did not inhibit or potentiate the chemotherapeutic activity of DA based on the evaluated parameters. Similarly, we did not observe relapse of parasitemia 20 days post-treatment in the DA alone or combination with AR-treated rats. Therefore, AR appears to be less effective in the treatment of trypanosomosis due to *T. brucei* (Federer strain) compared to DA. However, there is a need to evaluate the efficacy of higher differential therapeutic doses of AR in *T. brucei*-infected rats in case a low dose might be responsible for the observed low efficacy.

## Authors’ Contributions

TNE: Conceptualization. TNE, SEA, NAS, OZT, and ISI: Preparation and experimentation. TNE, SEA, NAS, OZT, and ISI: Results collation, analysis, and interpretation. TNE: Drafted the manuscript. TNE, SEA, NAS, OZT, and ISI: Revision, vetting, and approval of the final manuscript for submission. All authors have read and approved the final manuscript.
